# Association between vitamin K_1_ intake and mortality in the Danish Diet, Cancer, and Health cohort

**DOI:** 10.1007/s10654-021-00806-9

**Published:** 2021-09-30

**Authors:** Claire R. Palmer, Jamie W. Bellinge, Frederik Dalgaard, Marc Sim, Kevin Murray, Emma Connolly, Lauren C. Blekkenhorst, Catherine P. Bondonno, Kevin D. Croft, Gunnar Gislason, Anne Tjønneland, Kim Overvad, Carl Schultz, Joshua R. Lewis, Jonathan M. Hodgson, Nicola P. Bondonno

**Affiliations:** 1 Institute for Nutrition Research, School of Medical and Health Sciences, Edith Cowan University, Perth, Australia; 2 School of Medicine, University of Western Australia, Perth, Australia; 3 Department of Cardiology, Royal Perth Hospital, Perth, Australia; 4 Department of Cardiology, Herlev & Gentofte University Hospital, Copenhagen, Denmark; 5 School of Population and Global Health, University of Western Australia, Perth, Australia; 6 School of Biomedical Sciences, University of Western Australia, Royal Perth Hospital, Rear 50 Murray St, Perth, WA 6000 Australia; 7 The National Institute of Public Health, University of Southern Denmark, Odense, Denmark; 8 The Danish Heart Foundation, Copenhagen, Denmark; 9 The Danish Cancer Society Research Centre, Copenhagen, Denmark; 10 Department of Public Health, University of Copenhagen, Copenhagen, Denmark; 11 Department of Public Health, Aarhus University, Aarhus, Denmark; 12 Aalborg University Hospital, Aalborg, Denmark; 13 Centre for Kidney Research, Children’s Hospital at Westmead, School of Public Health, Sydney Medical School, The University of Sydney, Sydney, NSW Australia

**Keywords:** Phylloquinone, Cardiovascular disease, Cancer, Prospective cohort study

## Abstract

Reported associations between vitamin K_1_ and both all-cause and cause-specific mortality are conflicting. The 56,048 participants from the Danish Diet, Cancer, and Health prospective cohort study, with a median [IQR] age of 56 [52–60] years at entry and of whom 47.6% male, were followed for 23 years, with 14,083 reported deaths. Of these, 5015 deaths were CVD-related, and 6342 deaths were cancer-related. Intake of vitamin K_1_ (phylloquinone) was estimated from a food-frequency questionnaire (FFQ), and its relationship with mortality outcomes was investigated using Cox proportional hazards models. A moderate to high (87–192 µg/d) intake of vitamin K_1_ was associated with a lower risk of all-cause [HR (95%CI) for quintile 5 vs quintile 1: 0.76 (0.72, 0.79)], cardiovascular disease (CVD)-related [quintile 5 vs quintile 1: 0.72 (0.66, 0.79)], and cancer-related mortality [quintile 5 vs quintile 1: 0.80 (0.75, 0.86)], after adjusting for demographic and lifestyle confounders. The association between vitamin K_1_ intake and cardiovascular disease-related mortality was present in all subpopulations (categorised according to sex, smoking status, diabetes status, and hypertension status), while the association with cancer-related mortality was only present in current/former smokers (p for interaction = 0.002). These findings suggest that promoting adequate intakes of foods rich in vitamin K_1_ may help to reduce all-cause, CVD-related, and cancer-related mortality at the population level.

**Supplementary Information:**

The online version contains supplementary material available at 10.1007/s10654-021-00806-9.

## Introduction

Vitamin K, a group of fat-soluble vitamins, is important for blood clotting, as well as bone and vascular health and other physiological functions [1]. In the body vitamin K act as an essential cofactor for the γ-carboxylation of glutamate residues on vitamin K-dependant proteins [1]. There are two main forms of dietary vitamin K: vitamin K_1_ (phylloquinone) and vitamin K_2_ (menaquinones; MKs). Vitamin K_1_ is most abundant in green leafy vegetables and their oils, but is also present in smaller concentrations in the majority of food groups including fruit, meat and dairy products [2]. Studies on vitamin K absorption are lacking, but from the limited data available, the bioavailability of vitamin K_1_ from vegetables appears to be low (5–10%), with higher absorption observed from oil-based sources [3]. Vitamin K_2_ is a complex group of compounds differing from one another by the length of their side chain, which ranges from four to thirteen isoprenoid unit repeats (MK-4 to MK-13). MK-4 is found in animal products, such as meat and eggs, while all other MKs are typically found in fermented foods [3]. There is currently limited information available on the metabolism, absorption, bioavailability, and dietary sources of the different vitamin K_2_ isomers [4]. In addition, few food composition databases of vitamin K include all isoforms of vitamin K_2_, if they include vitamin K_2_ at all. As such, it is not currently possible to accurately estimate dietary intakes of vitamin K_2_, making it difficult to draw conclusions regarding its association with health outcomes in observational studies.

The effects of vitamin K on coagulation are well-established, and vitamin K is now recognized to be required for the prevention of osteoporotic bone loss and vascular calcification, implicated in the development of cardiovascular diseases [5]. There are numerous mechanisms by which vitamin K may moderate cardiovascular health; key pathways include the activation of the vitamin K-dependant protein, Matrix Gla Protein, a potent inhibitor of arterial calcification [6], and the reduction of systematic inflammation [7, 8]. Vitamin K_1_ is hypothesised to have anti-cancer effects and has been found to inhibit some human tumour cell lines, but the evidence is weak [9, 10]. Evidence from observational studies is limited and provides conflicting reports as to the association between vitamin K_1_ and both all-cause [11–14] and cause-specific mortality, including CVD- [11, 13, 14] and cancer-related mortality [11, 13, 15, 16]. The inconsistent findings between observational studies warrants further investigation of the potential associations in a large cohort setting.

Our primary aim was to investigate whether dietary intakes of vitamin K_1_ were associated with all-cause, CVD-related, or cancer-related mortality in the Danish Diet, Cancer, and Health cohort. Our secondary aim was to identify subpopulations that may benefit the most from higher vitamin K_1_ intakes.

## Methods

### Study population

The Danish Diet, Cancer, and Health cohort study recruited 57,053 participants from December 1993 to May 1997 who were between the ages of 50–65 years and who resided in the greater area of Copenhagen and Aarhus, Denmark. Of these, 56,468 were without cancer and completed a food frequency questionnaire (FFQ) at enrolment. Details of the study design, measurement procedures and participant characteristics for the Danish Diet, Cancer, and Health study have been previously published [17]. Crosslinking between the Danish Diet, Cancer, and Health cohort and nationwide registers was enabled by the unique and permanent civil registration numbers provided to all Danish residents. Five databases were linked to the cohort on the individual level: (1) The Civil Registration System which includes data on age, sex and vital status; (2) The Integrated Database for Labour Market Research [18] which provides information on annual income and employment status; (3) The Danish Register of Causes of Death contains information on the cause of death since 1994 by International Classification of Diseases (ICD) codes; (4) The Danish National Prescription Registry containing information on all filled prescriptions since 1994; (5) The Danish National Patient Register (DNPR) provides data on all hospital admissions in Denmark since 1978 with all diagnoses defined by ICD codes. The 8th revision of the ICD (ICD-8) was used until 1993 and the 10^th^ revision (ICD-10) from 1994 onwards. Participants were excluded from analyses in the present study if their data was missing or implausible (n = 215), or if they had extreme energy intakes [< 2,092 kJ/day (< 500 kcal/day) and > 20,920 kJ/day (> 5000 kcal/day)] (n = 205). This left 56,048 participants for analysis in the present study (Supplementary Fig. 1). This study was approved by the Danish Data Protection Agency (Ref no 2012–58-0004 I-Suite nr: 6357, VD-2018–117).

### Exposures

The exposures of interest in this study were intakes of vitamin K_1_ estimated from a 192-item semi-quantitative, self-administered FFQ, mailed to participants prior to their visit to one of two study centres. The FFQ has been validated for macro- and micro-nutrients against two times seven days of weighed diet records [19]. The FFQ asked participants to indicate their usual frequency of intake of different food and beverage items over the previous 12 months, using a 12-category frequency scale that ranged from never to eight times or more per day [17]. The intake of dietary vitamin K_1_ was estimated by multiplying the food/beverage item consumed (g/d) by the mean vitamin K_1_ value (μg/g) obtained from the Frida Food Data database [20] and, when a value was not available, the United States Department of Agriculture (USDA) database [21], as described previously [22].

### Study outcomes

The National Death Register was used to obtain the cause of death, while the date of death and vital status were obtained from the Civil Registration System for each participant. Cardiovascular disease-related mortality was defined as any ICD-10 diagnosis registered as a cause of death related to CVD (I00-I99) and cancer-related mortality was defined as any ICD-10 diagnosis registered as a cause of death related to cancer (C00-C99), dated after participant enrolment (Supplementary Table 1).

### Covariates

Self-administered questionnaires were used to obtain information on sex, age, education, smoking habits, alcohol consumption, and physical activity [23] upon enrolment into the study. Anthropometric and clinical measurements, such as BMI or total blood cholesterol, were taken at the study centres. Annual income, used as a proxy for socio-economic status, was defined as household income after taxation and interest for the value of the Danish currency in 2015. Income was estimated as the mean income over 5 years, up to and including the year of study inclusion, divided into quartiles. ICD-8 and ICD-10 codes (Supplementary Table 1) were used to determine the presence of prevalent chronic kidney disease (CKD), chronic obstructive pulmonary disease (COPD), ischemic heart disease (IHD), peripheral artery disease (PAD), ischemic stroke, heart failure, atrial fibrillation, and cancer at baseline. Prevalent CVD was defined by the presence of at least one diagnosis of IHD, PAD, ischemic stroke, heart failure, or atrial fibrillation prior to recruitment. For diabetes mellitus, only self-reported data were used due to the low validity of ICD-codes in the DNPR^27^. The use of antihypertensive medications or statin therapy at study enrolment were determined from the combination of Anatomical Therapeutic Chemical (ATC) codes in the Danish National Prescription Registry and self-reported use. Presence of hypercholesterolemia was defined using self-reported data. The presence of hypertension at baseline was determined through self-reported data or if ≥ 2 prescriptions of antihypertensive medication (ATC codes described in Supplementary Table 2) were claimed within 180 days prior to study enrolment [24]. The use of vitamin K antagonists (VKA) prior to baseline were also determined through ATC codes (ATC code: B01AA).

### Statistical analysis

Participants were followed for a maximum of 23 years, from the date of enrolment until the date of death, emigration, or end of follow-up (August, 2017), whichever was the first occurrence. Correlations between vitamin K_1_ intake and vegetable intake were examined using Spearman’s correlation coefficients. Vitamin K_1_ intake was categorized into quintiles of intake (i.e. 20% of participants from the total study population in each category) for the purpose of presentation, but not for modelling. Potential nonlinear relationships were examined using restricted cubic splines, with hazard ratios (HRs) obtained from Cox proportional hazards models, using the ‘rms’ R package with the rcs() function [25]. HRs with 95% confidence intervals (CIs) were plotted for each unit of vitamin K_1_ intake (continuous), with the median vitamin K_1_ intake in quintile 1 as the reference level. For visual simplicity, the x-axes of all cubic splines were restricted to be within three standard deviations of the mean. The test of nonlinearity used a chi-squared test to compare the model with only the linear term to the model that included the cubic spline terms. Cox proportional hazards assumptions were tested using log–log plots of the survival function vs. time which were assessed for parallel appearance, with no violations found. Four models of adjustment were used: (1a) minimally adjusted; (1b) multivariable-adjusted; (2) multivariable-adjusted including potential dietary confounders that are not also a major dietary source of the exposure; (3) exploratory model including covariates in model 2 plus major dietary sources of the exposure (information of specific covariates used is presented in the relevant table and figure legends as well as in Supplementary Table 3). Covariates were chosen a priori using knowledge of potential confounders of vitamin K_1_ intake and either CVD- or cancer-related mortality. In a sensitivity analysis, participants were excluded if they were prescribed VKA at baseline (n = 319) and censored if they were prescribed a VKA during follow-up (n = 6690). To investigate potential effect modification, analyses were stratified by subgroups with differing risk of CVD and/or cancer, namely, sex, baseline smoking status (ever versus never smokers), diabetes status, and presence of hypertension. We tested for interactions using a chi-squared test comparing nested models. To identify subpopulations that may benefit the most from higher vitamin K_1_ intakes, standard logistic regression models were used to obtain the 20-year absolute risk estimates of mortality. For these analyses, a binary outcome indicating death during 20 years of follow-up was used. Unless indicated by the stratification variable, these estimates are for an ‘average’ non-smoking cohort participant, aged 56 years, with a BMI of 25.5 kg/m^2^, a total daily metabolic equivalent score of 56, with a mean household income of 394,701–570,930 DKK/year, 8–10 years of education, an alcohol intake of 13 g/day, and no prevalent chronic disease at baseline (diabetes, cancer, chronic obstructive pulmonary disease, or chronic kidney disease). To investigate whether associations between vitamin K_1_ and mortality outcomes were evident amongst participants with different levels of vegetable consumption, we stratified our primary analysis by tertiles of total vegetable intake.

Analyses were undertaken using STATA/IC (14.2 StataCorp LLC) and R statistics (R Core Team, 2019) [26].

## Results

### Baseline characteristics

The cohort, comprised of 56,048 Danish residents, had a median [IQR] age of 56 [52–60] years at entry and were followed up for 21 [20–22] years. During the 1,085,186 person-years of follow-up, 14,083 participants died from any cause. Of these, 5015 deaths were CVD-related, and 6342 deaths were cancer-related. The baseline characteristics of the study population, overall and stratified by vitamin K_1_ and vitamin K_2_ intake quintiles are shown in Table 1.

**Table 1:** Baseline characteristics of study population by vitamin K_1_ intake quintiles

	Total population	Vitamin K_1_ intake quintiles				
	n = 56,048	Q1 n = 11,210	Q2 n = 11,209	Q3 n = 11,210	Q4 n = 11,209	Q5 n = 11,210
*Demographics*						
Total vitamin K_1_ intake (µg/d)	113 [80–151]	57 [47–66]	87 [80–94]	113 [107–120]	142 [134–151]	192 [174–219]
Sex (male)	26,666 (47.6)	4876 (43.5)	5119 (46.5)	5210 (46.5)	5495 (49.0)	5966 (53.2)
Age (years)	56 [52–60]	56 [53–60]	56 [52–60]	56 [52–60]	56 [52–60]	56 [52–60]
BMI (kg/m^2^)	26 [9, 23–27]	26 [9, 10, 24–27]	26 [9, 24–27]	26 [9, 23–27]	25 [9, 23–27]	25 [9, 23–27]
MET score	57 [37–85]	49 [31–76]	54 [35–82]	57 [38–84]	59 [40–86]	64 [42–93]
*Smoking status*						
Never	19,666 (35.1)	3241 (28.9)	3798 (33.9)	4183 (37.3)	4299 (38.4)	4145 (37.0)
Former	16,153 (28.8)	2,703 (24.1)	3,145 (28.1)	3,226 (28.8)	3,427 (30.6)	3,652 (32.6)
Current	20,229 (36.1)	5266 (47.0)	4266 (38.1)	3801 (33.9)	3483 (31.1)	3413 (30.4)
Smoking pack-years	9 [0–26]	19 [0–32]	12 [0–28]	8 [0–25]	7 [0–23]	5 [0–21]
Education						
≤ 7 years	18,466 (32.9)	4818 (43.0)	4146 (37.0)	3673 (32.8)	3193 (28.5)	2636 (23.5)
8–10 years	25,817 (46.1)	5038 (44.9)	5271 (47.0)	5336 (47.6)	5205 (46.4)	4967 (44.3)
≥ 11 years	11,737 (20.9)	1345 (12.0)	1790 (16.0)	2199 (19.6)	2804 (25.0)	3599 (32.1)
*Annual income*						
≤ 394,700 DKK/year	13,919 (24.8)	3680 (32.8)	2829 (25.2)	2580 (23.0)	2395 (21.4)	2435 (21.7)
394,701–570,930 DKK/year	14,018 (25.0)	3097 (27.6)	2979 (26.6)	2895 (25.8)	2545 (22.7)	2502 (22.3)
570,931–758,297 DKK/year	14,054 (25.1)	2584 (23.1)	2921 (26.1)	2966 (26.5)	2964 (26.4)	2619 (23.4)
> 758,297 DKK/year	14,057 (25.1)	1849 (16.5)	2480 (22.1)	2769 (24.7)	3305 (29.5)	3654 (32.6)
Hypertensive	9428 (16.8)	2027 (18.1)	1916 (17.1)	1910 (17.0)	1833 (16.4)	1742 (15.5)
Hypercholesterolemic	4,193 (7.5)	846 (7.5)	830 (7.4)	885 (7.9)	839 (7.5)	793 (7.1)
*Comorbidities*						
Diabetes	1182 (2.1)	197 (1.8)	188 (1.7)	205 (1.8)	229 (2.0)	363 (3.2)
COPD	858 (1.5)	230 (2.1)	180 (1.6)	141 (1.3)	163 (1.5)	144 (1.3)
CKD	204 (0.4)	29 (0.3)	55 (0.5)	46 (0.4)	40 (0.4)	34 (0.3)
Cancer	247 (0.4)	66 (0.6)	44 (0.4)	49 (0.4)	43 (0.4)	45 (0.4)
Heart failure	220 (0.4)	63 (0.6)	43 (0.4)	28 (0.2)	48 (0.4)	38 (0.3)
Atrial fibrillation	451 (0.8)	95 (0.8)	83 (0.7)	86 (0.8)	86 (0.8)	101 (0.9)
IHD	2,200 (3.9)	524 (4.7)	465 (4.1)	396 (3.5)	400 (3.6)	415 (3.7)
PAD	498 (0.9)	145 (1.3)	124 (1.1)	85 (0.8)	82 (0.7)	62 (0.6)
Stroke	787 (1.4)	201 (1.8)	177 (1.6)	132 (1.2)	142 (1.3)	135 (1.2)
*Medication use*						
Insulin treated	695 (1.2)	103 (0.9)	105 (0.9)	111 (1.0)	132 (1.2)	244 (2.2)
Antihypertensive	6904 (12.7)	1482 (13.2)	1413 (12.6)	1392 (12.4)	1350 (12.0)	1267 (11.3)
Statin	1101 (2.0)	221 (2.0)	238 (2.1)	225 (2.0)	208 (1.9)	209 (1.9)
HRT (% of females)						
Never	15,972 (54.4)	3214 (20.1)	3064 (19.2)	3324 (20.8)	3232 (20.2)	3138 (19.6)
Current	8825 (30.0)	1736 (19.7)	1781 (20.2)	1771 (20.1)	1730 (19.6)	1807 (20.5)
Former	4553 (15.5)	1010 (22.2)	841 (20.3)	841 (18.5)	885 (19.4)	895 (19.7)
NSAID	18,161 (32.6)	3656 (32.9)	3689 (33.1)	3621 (32.5)	3647 (32.7)	3548 (31.8)
Aspirin	7,097 (12.7)	1,515 (13.5)	1,420 (12.7)	1,415 (12.6)	1,346 (12.0)	1,401 (12.5)
*Dietary characteristics*						
Energy (kJ)	9500 [7858–11,369]	7697 [6461–9,210]	8846 [7512–10,382]	9544 [8,159–11,127]	10,255 [8760–11,966]	11,292 [9630–13,235]
Total fish intake (g/d)	38 [25–55]	28 [18–41]	34 [23–49]	38 [27–54]	43 [30–60]	50 [34–70]
Red meat intake (g/d)	78 [57–107]	67 [49–90]	76 [56–102]	81 [59–108]	83 [61- 113]	87 [61–121]
Processed meat intake (g/d)	25 [14–40]	23 [13–36]	25 [15–39]	25 [15–40]	25 [15–42]	26 [14–43]
Fruit intake (g/d)	171 [94–281]	109 [50–188]	148 [82–238]	175 [103–277]	200 [123–313]	241 [146–381]
Vegetable intake (g/d)	161 [104–231]	77 [53–108]	128 [94–166]	167 [127–211]	203 [158–256]	270 [206–345]
Wholegrain intake (g/d)	128 [86–175]	88 [65–125]	117 [81–164]	129 [92–171]	153 [110–194]	172 [123–224]
Alcohol intake (g/d)	13 [6–29]	12 [4–30]	12 [6–29]	13 [6–28]	14 [7–29]	14 [7–29]

Data expressed as median [IQR] or n (%), unless otherwise stated

BMI, body mass index; CKD, chronic kidney disease; COPD, common obstructive pulmonary disease; DKK, Danish Krone; HRT, hormone replacement therapy, MET, metabolic equivalent; NSAID, Nonsteroidal anti-inflammatory drug

At baseline, the median intake [IQR] of vitamin K_1_ was 113.3 µg/day [80.2–150.6]. There was a strong correlation between vitamin K_1_ and vegetable intake (Spearman’s rho = 0.82, *p* < 0.001). Compared to participants in the lowest quintile of vitamin K_1_ intake, a greater percentage of participants in the highest quintile were male, more physically active, had a higher level of education, had a higher income, had never smoked, and had a higher energy intake and a higher intake of alcohol, fish, red meat, wholegrains, fruits and vegetables. Furthermore, they had a higher prevalence of diabetes, but a lower prevalence of hypertension, COPD, IHD, PAD, stroke, and heart failure.

### Associations between vitamin K_1_ intake and all-cause mortality

**Fig. 1:**
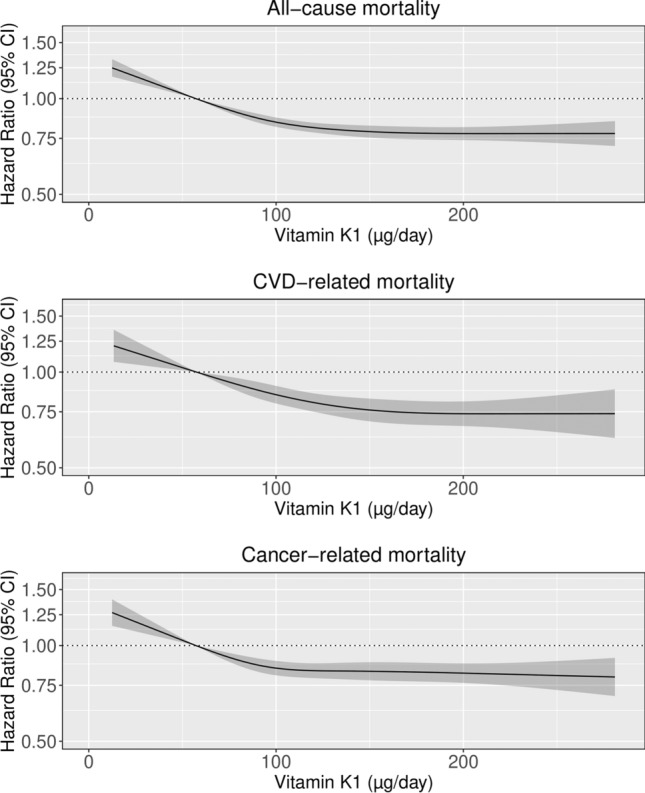
Hazard ratios from Cox proportional hazards model with restricted cubic spline curves describing the association between vitamin K_1_ intake (µg/day) and all-cause mortality, cardiovascular disease (CVD)-related mortality and cancer-related mortality. Hazard ratios are based on models adjusted for age, sex, BMI, smoking status, smoking pack-years, social economic status (income), physical activity, alcohol intake, and education (Model 1b), and are comparing the specific level of vitamin K_1_ intake (horizontal axis) to the median intake for participants in the lowest intake quintile (57 µg/day)

The association between vitamin K_1_ intake and all-cause mortality was non-linear (p for non-linearity < 0.001); the gradient of the inverse association started to decrease at intakes above approximately 100 µg/day (Fig. 1). The HRs for all-cause and cause-specific mortality, by quintiles of vitamin K_1_ intake, are shown in Table 2. Participants with the highest intakes of vitamin K_1_ (quintile 5), had the lowest risk of all-cause mortality relative to participants in quintile 1 [HR: 0.78 (0.74, 0.81); Model 1b]. There was a clear relationship between vitamin K_1_ intake and all-cause mortality after adjusting for potential dietary confounders (Model 2).

**Table 2:** Hazard ratios all-cause and cause-specific mortality by quintiles of vitamin K_1_ intake

	Vitamin K_1_ intake quintiles				
	Q1 n = 11,210	Q2 n = 11,209	Q3 n = 11,210	Q4 n = 11,209	Q5 n = 11,210
Intake (mg/d)*	57 [4–73]	87 [73–100]	113 [100–127]	142 [127–161]	192 [161–800]
*All-cause mortality*					
No. events	3754	3034	2582	2431	2282
HR (95% CI)					
Model 1a	Ref	0.76 (0.74, 0.79)	0.66 (0.64, 0.68)	0.61 (0.59, 0.63)	0.58 (0.56, 0.61)
Model 1b	Ref	0.88 (0.85, 0.90)	0.82 (0.79, 0.85)	0.79 (0.76, 0.83)	0.78 (0.74, 0.81)
Model 2	Ref	0.86 (0.84, 0.89)	0.81 (0.78, 0.84)	0.78 (0.75, 0.82)	0.77 (0.73, 0.81)
Model 3	Ref	0.86 (0.83, 0.89)	0.81 (0.77, 0.84)	0.78 (0.74, 0.83)	0.77 (0.71, 0.83)
*CVD-related mortality*					
No. events	1364	1157	885	839	768
HR (95% CI)					
Model 1a	Ref	0.76 (0.72, 0.81)	0.65 (0.61, 0.69)	0.58 (0.54, 0.62)	0.54 (0.50, 0.59)
Model 1b	Ref	0.88 (0.84, 0.94)	0.81 (0.76, 0.87)	0.77 (0.71, 0.83)	0.74 (0.68, 0.81)
Model 2	Ref	0.86 (0.81, 0.92)	0.79 (0.74, 0.85)	0.75 (0.68, 0.82)	0.72 (0.64, 0.80)
Model 3	Ref	0.87 (0.82, 0.93)	0.81 (0.74, 0.88)	0.77 (0.69, 0.87)	0.75 (0.65, 0.88)
*Cancer-related mortality*					
No. events	1644	1325	1176	1170	1027
HR (95% CI)					
Model 1a	Ref	0.78 (0.74, 0.81)	0.69 (0.66, 0.73)	0.66 (0.62, 0.70)	0.63 (0.59, 0.67)
Model 1b	Ref	0.88 (0.84, 0.92)	0.84 (0.79, 0.88)	0.83 (0.78, 0.88)	0.82 (0.77, 0.88)
Model 2	Ref	0.86 (0.82, 0.91)	0.82 (0.78, 0.87)	0.82 (0.76, 0.88)	0.81 (0.74, 0.88)
Model 3	Ref	0.87 (0.82, 0.91)	0.82 (0.77, 0.88)	0.82 (0.75, 0.90)	0.81 (0.72, 0.92)

Hazard ratios (95% Confidence Intervals) for all-cause and cause-specific mortality during 23 years of follow-up, obtained from restricted cubic splines based on Cox proportional hazards models. Model 1a adjusted for age and sex; Model 1b adjusted for age, sex, BMI, smoking status, smoking pack-years, physical activity, alcohol intake, social economic status (income), education, and prevalent disease; Model 2 adjusted for all covariates in Model 1b plus energy intake and intakes of fish, red meat, processed meat, wholegrains, and fruit; Model 3 adjusted for all of the covariates in Model 2 plus intake of vegetables

*Median; range in parentheses (all such values)

### Associations between vitamin K_1_ intake and CVD-related mortality

The association between vitamin K_1_ intake and CVD-related mortality was non-linear (p for non-linearity < 0.001; Fig. 1). Relative to participants in quintile 1 and after adjusting for demographic and lifestyle confounders, participants in quintile 5 had the lowest risk of CVD-related mortality [HR: 0.74 (0.68, 0.81); Model 1b; Table 2]. A clear inverse association remained after further adjustments for dietary confounders (Models 2 and 3; Table 2).

### Associations between vitamin K_1_ intake and cancer-related mortality

The association between vitamin K_1_ intake and cancer-related mortality was non-linear (p for non-linearity < 0.01; Fig. 1). Relative to participants in quintile 1 and after adjusting for demographic and lifestyle confounders, participants in quintile 5 had the lowest risk of cancer-related mortality [HR: 0.82 (0.77, 0.88); Model 1b; Table 2]. A clear inverse relationship remained after adjusting for potential dietary confounders (Model 2) and vitamin K_1_ intake (Model 3), although the point estimates levelled beyond moderate intakes (quintile 3).

### Sensitivity analysis

The exclusion of participants prescribed a VKA (n = 7,009) did not change the relationship of vitamin K_1_ intake with mortality outcomes (Supplementary Fig. 2).

### Stratified analysis

**Fig. 2:**
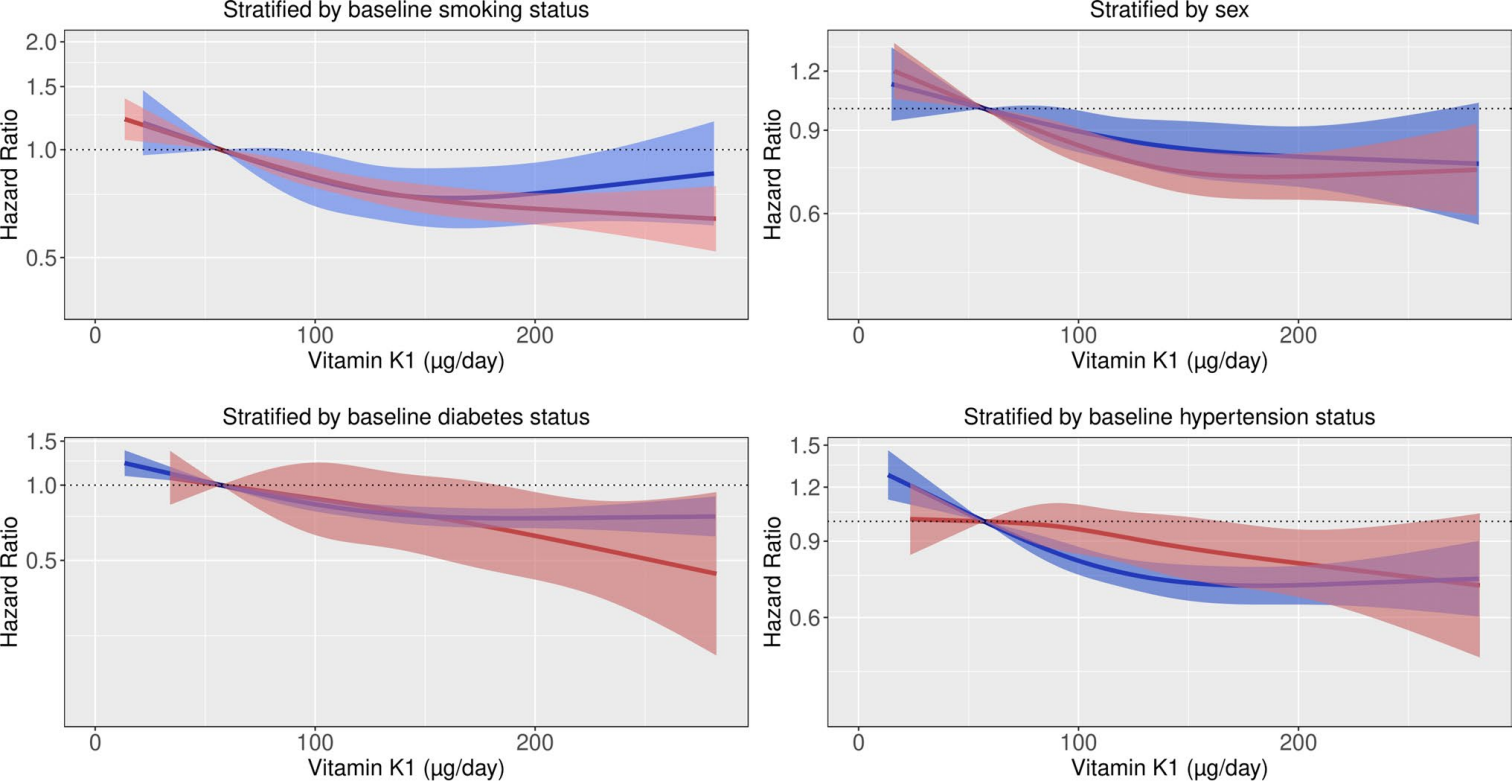
Hazard ratios from Cox proportional hazards model with restricted cubic spline curves describing the association between vitamin K_1_ intake (µg/day) cardiovascular disease (CVD)-related mortality, stratified by baseline smoking status, smoking pack-years, sex, baseline diabetes status, and baseline hypertension status. Hazard ratios are based on models adjusted for age, sex, BMI, smoking status, social economic status (income), physical activity, alcohol intake, and education (Model 1b), and are comparing the specific level of vitamin K_1_ intake (horizontal axis) to the median intake for participants in the lowest intake quintile (57 µg/day)

The association between vitamin K_1_ intake and CVD-related mortality was present in all subgroups investigated (p for interaction > 0.05 for all; Fig. 2). However, as the incidence of CVD-mortality was higher in smokers, and those with diabetes, the absolute difference (vitamin K_1_ intake quintile 5 – vitamin K_1_ intake quintile 1) in the 20-year estimated risk of CVD-mortality was greater in these subgroups [current smokers (2.53% for males, 1.43% for females); persons with diabetes (2.46% for males, 1.39% for females); Supplementary Table 4].

**Fig. 3:**
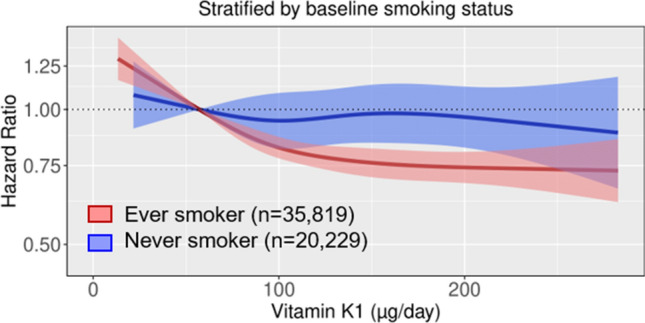
Hazard ratios from Cox proportional hazards model with restricted cubic spline curves describing the association between vitamin K_1_ intake (µg/day) cancer-related mortality, stratified by baseline smoking status. Hazard ratios are based on models adjusted for age, sex, BMI, smoking pack-years, social economic status (income), physical activity, alcohol intake, and education (Model 1b), and are comparing the specific level of vitamin K_1_ intake (horizontal axis) to the median intake for participants in the lowest intake quintile (57 µg/d)

The association between vitamin K_1_ intake and cancer-related mortality was only present in current/former smokers (p for interaction = 0.002; Fig. 3). As cancer-mortality incidence was higher in current smokers, the absolute difference (vitamin K_1_ intake quintile 5 – vitamin K_1_ intake quintile 1) in the 20-year estimated risk of cancer-mortality was the largest in this subgroup [current smokers (3.51% males, 2.96% females); Supplementary Table 5].

To investigate whether vitamin K_1_ is just a marker of higher vegetable intake, due to the high correlation between vitamin K_1_ intake and vegetable intake, we 1) adjusted for total vegetable intake in Model 3, and 2) we stratified our primary analysis by tertiles of total vegetable intake. Adjusting for total vegetable intake did not materially alter the observed associations (Table 2). Furthermore, there was evidence of an inverse association between vitamin K_1_ intakes and all-cause mortality in all tertiles of total vegetable intake (Model 1b; Supplementary Table 6).

## Discussion

In this large prospective cohort study of 56,048 Danish men and women, a moderate to high intake of vitamin K_1_ was associated with a lower risk of all-cause mortality, CVD-related mortality, and cancer-related mortality, after adjusting for potential lifestyle confounders. On a relative scale, the association between vitamin K_1_ and CVD-related mortality was not modified by sex or smoking, diabetes and hypertension status. However, as CVD-mortality incidence was higher in smokers, and those with diabetes, the absolute difference in the 20-year estimated risk of CVD-mortality was greater in these subgroups. The association between vitamin K_1_ intakes and cancer-mortality was only present in current and former smokers.

To our knowledge, four epidemiological studies have investigated the associations between vitamin K_1_ intake and all-cause mortality, two of which reported no association [11, 12]. Notably, Zwakenberg et al*.* reported relatively high vitamin K_1_ intakes in the EPIC-Netherlands cohort [11] which may explain the lack of an association; the mean intake in the lowest energy-adjusted quartile was estimated to be 87.7 ± 17.5 µg/d which is higher than the recommended adequate intake of 70 µg/d of vitamin K_1_ set by the European Food Safety Authority [4]. In the present study, we saw a threshold at approximately 100 µg/d of vitamin K_1_ (equivalent to approximately 1 cup of leafy green vegetables), after which there appeared to be minimal additional benefit. As such, participants of the EPIC-NL cohort in the lowest intake quartile may be a poor reference to observe inverse associations due to their diet likely containing adequate vitamin K_1_. Similarly, the study by Geleijnse et al. also had a high vitamin K_1_ intake in their reference group, with a median energy-adjusted lower tertile of 154.6 µg/d [12]. The other two studies reported a significant inverse association between vitamin K_1_ status and all-cause mortality [13, 14]. The study by Shea et al. measured circulating phylloquinone concentrations, which has been shown to associate with dietary phylloquinone intake up to intakes of 200 µg/d [27], and found that participants with low circulating phylloquinone concentrations (≤ 0.5 nmol/L) had a 19% higher risk of mortality compared to participants with higher circulating phylloquinone concentrations (> 1.0 nmol/L) [fully adjusted HR: 1.19 (1.03, 1.38)] [14]. Similarly, Juanola-Falgarona et al*.* reported that an increase in energy-adjusted vitamin K_1_ intake during follow-up was associated with a 36% reduced risk of all-cause mortality in a Mediterranean population at high CVD risk [fully adjusted HR for highest compared to lowest intakes: 0.64 (0.45, 0.90)] [13]. The aforementioned findings support those of the current study where participants in the highest quintile of vitamin K_1_ intake had a 16% lower risk of mortality from any cause.

There are few studies to date that have examined associations between vitamin K_1_ and CVD mortality. Of the studies available, all reported no associations between CVD mortality and both vitamin K_1_ intake [11, 13] and circulating levels of vitamin K_1_ [14]. In the present study, higher vitamin K_1_ intakes were associated with a lower risk of CVD-related mortality. Interestingly, we have reported previously in this cohort [22] that higher vitamin K_1_ intakes were independently associated with a 21% lower risk of an atherosclerotic CVD-related hospitalisation [multivariable adjusted HR for highest compared to lowest intakes: 0.79, 95% CI (0.74, 0.84)].

There is weak evidence from in vitro studies for the anti-carcinogenic activity of vitamin K_1_ [9, 10]. We found that a moderate to high intakes of vitamin K_1_ was associated with a lower risk of cancer-related mortality. Four prior epidemiological studies that examined cancer mortality and vitamin K_1_ intake are conflicted – three found no statistically significant association [11, 15, 16], and one reported an inverse association in an elderly Mediterranean population [13]. Conflicting findings between cohorts may be attributed to different diets, the use of different vitamin K_1_ food databases and/or differences in the inclusion of vitamin K-rich foods in the respective FFQs. However, in order to discern the relationship between vitamin K intake and cancer mortality, more studies are needed in additional cohorts with analysis of specific cancer subtypes, due to the complex nature of cancer.

Vegetables are the major dietary source of vitamin K_1_ and previous studies have demonstrated an inverse association between vegetable intake and mortality from any cause [28–30]. This raises the query of whether higher vitamin K_1_ intakes are simply a marker of higher vegetable intake and higher diet quality, and whether components other than vitamin K_1_ are responsible for the observed associations [13]. Although disentangling dietary components in observational studies is not possible, in the present study associations between vitamin K_1_ intakes and all-cause mortality remained after adjustments were made for potential dietary confounders, including total vegetable intake, as well as within participants in the highest vegetable intake tertile. This suggests vitamin K_1_ may provide additional benefits, beyond other components of a vegetable-rich diet.

In the present study, associations between total vitamin K_1_ intakes and CVD-mortality were present in all subpopulations investigated, although the number of participants with diabetes at baseline was low (n = 1,182), which explains the larger confidence intervals observed for this group. Interestingly, the association between vitamin K_1_ intake and cancer-mortality was only present in current/former smokers. We report that the difference in the 20-year estimated absolute risk of mortality was greatest for subgroups at the highest risk of CVD-mortality (current smokers and those with diabetes) and cancer-mortality (current smokers). Thus, if the association between higher vitamin K_1_ intakes and mortality is truly causal, ensuring adequate intakes of vitamin K_1_-rich foods in smokers and persons with diabetes could reduce mortality and disease burden on a population level. However, our data does not suggest the negative impact of smoking on health is negated by higher vitamin K intake, as smokers with higher vitamin K_1_ intakes remain at a much higher risk of both CVD- and cancer-related mortality than non-smokers at any level of vitamin K_1_ intake.

There are many strengths in the present study, including a long follow-up period of 23 years with a large adult population. Another strength is the crosslinking with Danish registries, which allowed for a negligible loss to follow up, aided capture of mortality outcomes and participant characteristics gathered at baseline, and enabled censoring of participants who were prescribed VKA at any point during follow up. We also examined whether vitamin K_1_ is simply a marker of vegetable intake. Acknowledging the limitations of the study is crucial for interpretation of findings; unfortunately, there was only one FFQ, administered at baseline and in the long follow-up period dietary changes were unaccounted for, which may have attenuated the power to detect an association. Additionally, this FFQ has not been validated for vitamin K, however previous studies have shown that plasma phylloquinone positively associates with phylloquinone intakes up to 200 µg/day, after which the association plateaued, likely attributable to over-reporting of vegetable intakes [27]. This may explain, at least in part, the plateau observed in the present study but, as intake estimates were derived from an FFQ, caution should be taken not to extrapolate absolute intakes. Secondly, due the lack of comprehensive vitamin K_2_ nutrient databases, we did not investigate associations with intakes of vitamin K_2_. Thirdly, the Danish population the cohort was drawn from is more ethnically and racially homogeneous than most countries, which may limit the generalisability of the findings. Fourthly, the coding of CVD-related and cancer-related deaths by the Danish Register of Causes of Death has not been validated and may be susceptible to misclassification bias. Most importantly, due to the observational nature of this work, causality cannot be inferred.

In this prospective cohort study, dietary vitamin K_1_ intake was inversely related to CVD-related mortality, cancer-related mortality, and mortality from any cause. Ensuring adequate intakes of vitamin K_1_, commonly found in foods such as green leafy and cruciferous vegetables, especially in high-risk individuals, may help to reduce CVD-related and cancer-related mortality at the population level.

## Supplementary Information

Below is the link to the electronic supplementary material.

Supplementary file1 (DOCX 208 kb)

## Declarations

### Availability of data and material

Data described in the manuscript, code book, and analytic code will be made available upon request pending application and approval by the Diet, Cancer and Health Steering Committee at the Danish Cancer Society.

### Code availability

Data described in the manuscript, code book, and analytic code will be made available upon request pending application and approval by the Diet, Cancer and Health Steering Committee at the Danish Cancer Society.
