# Supplementary material for: Association between vitamin K1 intake and mortality in the Danish Diet, Cancer, and Health cohort
Source: Eur J Epidemiol. 2021 Sep 30;36(10):1005–14. doi: 10.1007/s10654-021-00806-9 (PMC8542554; doi:10.1007/s10654-021-00806-9)
Supplement: Supplementary file 1 — Supplementary file1 (DOCX 208 kb) [file 10654_2021_806_MOESM1_ESM.docx]

**Supplementary Material**

**Association between vitamin K intake and mortality in the Danish Diet, Cancer, and Health cohort**

***European Journal of Epidemiology***

Claire R. Palmer, Jamie W. Bellinge, Frederik Dalgaard, Marc Sim, Kevin Murray, Emma Connolly, Lauren C. Blekkenhorst, Catherine P. Bondonno, Kevin D. Croft, Gunnar Gislason, Anne Tjønneland, Kim Overvad, Carl Schultz, Joshua R. Lewis, Jonathan M. Hodgson, Nicola P. Bondonno.

*Corresponding author: Nicola P Bondonno

Email: [n.bondonno@ecu.edu.au](about:blank)

**Supplementary Figures**

**
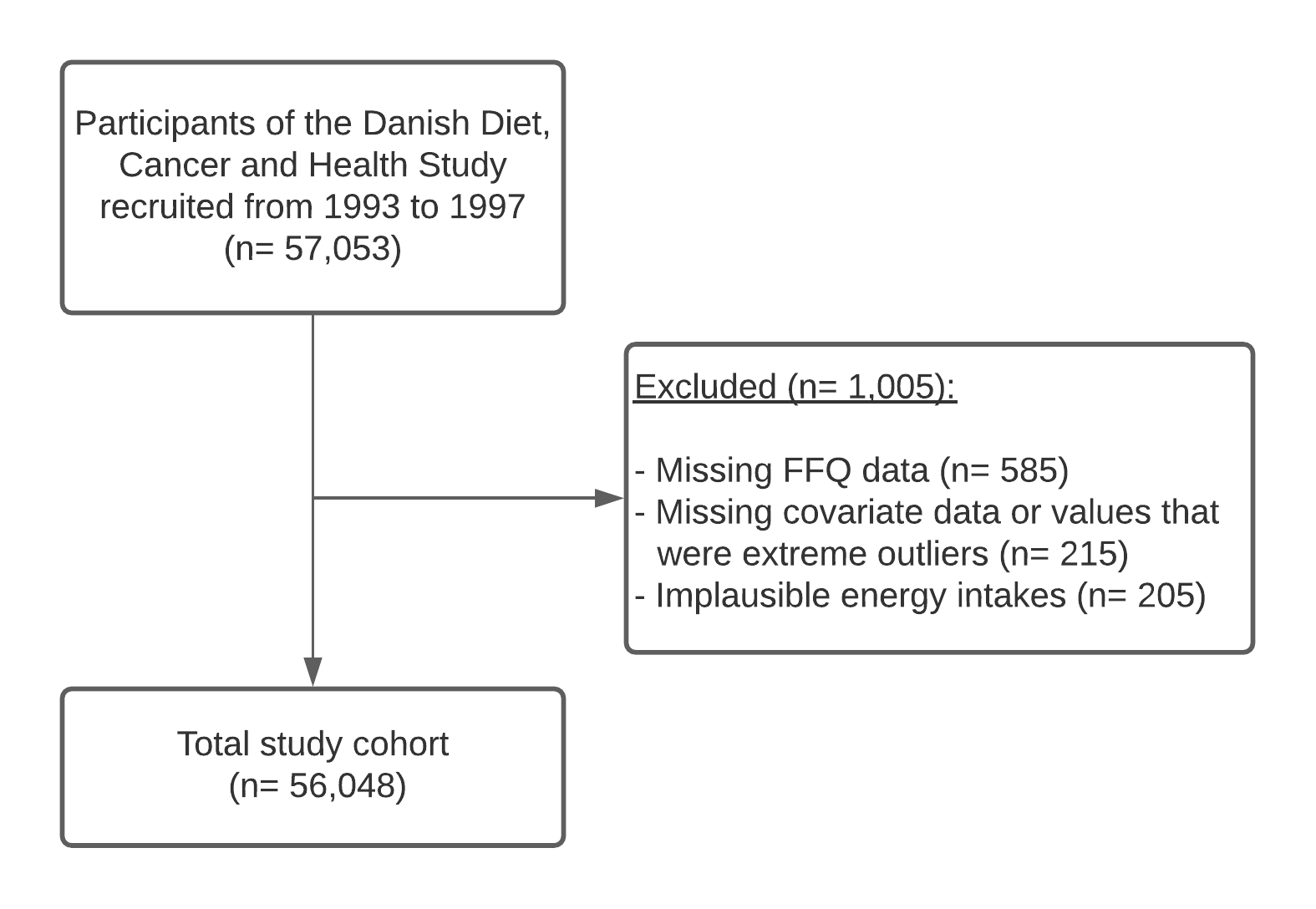
**

**Supplementary Figure 1.** Consort flow diagram

FFQ, food frequency questionaire

**
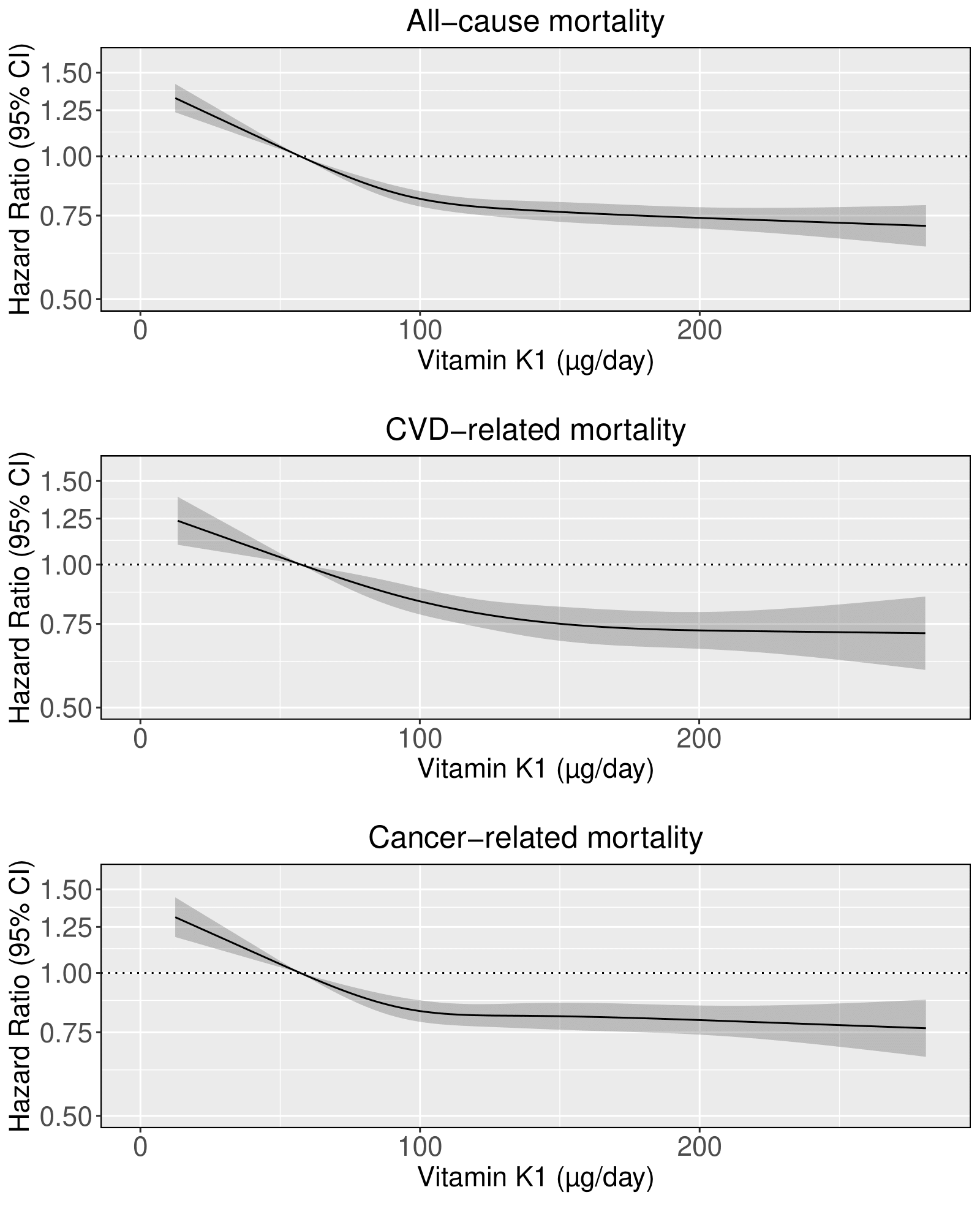
**

**Supplementary Figure 2.** The association between vitamin K_1_ intake (µg/day) and all-cause mortality, cardiovascular disease (CVD)-related mortality and cancer-related mortality, after censoring participants upon prescription of a vitamin K antagonist. Hazard ratios are derived from a Cox proportional hazards model with restricted cubic spline curves adjusting for age, sex, BMI, smoking status, smoking pack-years, social economic status (income), physical activity, alcohol intake, and education (Model 1b), and are comparing the specific level of vitamin K_1_ intake (horizontal axis) to the median intake for participants in the lowest intake quintile (57 µg/day).

**Supplementary Tables**

| **Supplementary Table 1**. International Classification of Disease codes used to determine prevalent disease and cause specific mortality | |
| --- | --- |
| **Disease and cause specific mortality** | **ICD codes (ICD-8; ICD-10)** |
| Ischemic heart disease | 410-414; I20-I25 |
| Ischemic stroke | 433-434; I63 |
| Peripheral artery disease | 440-444; I70-I74 |
| Heart failure | 4270-4271; I42, I50, I110, J81 |
| Atrial fibrillation | 42793-42794; I48 |
| Chronic kidney disease | 580-584; N02-N08, N11-N12, N14, N18-N19, N26, N158-N160, N162-N164, N168, Q61, E102, E112, E132, E142, I120, M321B |
| Chronic obstructive pulmonary disease | 491-493; J42-J44 |
| Cancers | 140-209; C00-C99 |
| Cardiovascular disease related mortality | I00-I99 |
| Cancer-related mortality | C00-C99 |

| **Supplementary Table 2.**  Anatomical Therapeutic Chemical codes for medications | |
| --- | --- |
| Antihypertensive medication (defined by the usage of any combination of at least two of the seven different drugs classes at the same time) | Non-Loop: Thiazides C02L, C02DA, C07B, C07D, C09XA52, C03A, C03EA;  Low-ceiling diuretics (excl. thiazides): C03B, C03X, C07C, C08G, C09BA, C09DA; potassium-sparing agents (spiron): C03D, C03E, C03EB  Loop: high-ceiling diuretics (Loop) C03C, C03EB  Antiadrenergic agents: C02A, C02B, C02C  Beta-blockers: C07A, C07B, C07C, C07D, C07F  Vasodilators: C02DB, C02DD, C02DG  Calcium channel blockers: C08, C09BB, C09DB  Renin angiotensin system inhibitors and angiotensin II receptor blockers: C09AA, C09BA, C09BB, C09CA, C09DA, C09DB, C09XA02, C09XA52 |
| Statin therapy | HMG CoA reductase inhibitors: C10AA |

| **Supplementary Table 3.** Covariates included in models | |
| --- | --- |
| *Model 1a:*  Minimally-adjusted | Age and sex |
| *Model 1b:*  Multivariable-adjusted | Age, sex, BMI, smoking status (current/former/never), smoking pack-years, physical activity (total daily metabolic equivalent), pure alcohol intake (g/d), social economic status (income), education, and prevalent disease (diabetes, chronic obstructive pulmonary disease, chronic kidney disease, CVD, and cancer; entered into the model separately). |
| *Model 2:*  Multivariable-adjusted including energy intake and potential dietary confounders | Age, sex, BMI, smoking status (current/former/never), smoking pack-years, physical activity (total daily metabolic equivalent), pure alcohol intake (g/d), social economic status (income), education, prevalent disease (diabetes, chronic obstructive pulmonary disease, chronic kidney disease, CVD, and cancer; entered into the model separately), energy intake (kJ), and intakes (g/d) of fish, red meat, processed meat, wholegrains, and fruit |
| *Model 3:*  Multivariable-adjusted including energy intake and additional potential dietary confounders | Age, sex, BMI, smoking status (current/former/never), smoking pack-years, physical activity (total daily metabolic equivalent), pure alcohol intake (g/d), social economic status (income), education, prevalent disease (diabetes, chronic obstructive pulmonary disease, chronic kidney disease, CVD, and cancer; entered into the model separately), energy intake (kJ), and intakes (g/d) of fish, red meat, processed meat, wholegrains, fruit and vegetables. |

| **Supplementary Table 4.** 20-year predicted risk of cardiovascular disease-related mortality for males and females | | | |
| --- | --- | --- | --- |
|  | **Vitamin K_1_ intake** | | **Risk difference**  **(%)** |
|  | **Q1**  **Risk (95% CI)** | **Q5**  **Risk (95% CI)** |  |
| **Males** |  |  |  |
| Non-smoker | 4.80 (4.21, 5.46) | 3.69 (3.21, 4.23) | 1.11 |
| Former smoker | 5.76 (5.09, 6.51) | 4.44 (3.89, 5.06) | 1.32 |
| Current smoker | 11.58 (10.45, 12.82) | 9.05 (8.03, 10.18) | 2.53 |
| Statin user | 6.59 (4.93, 8.76) | 5.08 (3.78, 6.80) | 1.51 |
| Statin non-user | 4.78 (4.20, 5.45) | 3.67 (3.20, 4.21) | 1.11 |
| Persons with hypertension | 7.92 (6.87, 9.10) | 6.13 (5.28, 7.11) | 1.79 |
| Persons without hypertension | 4.32 (3.78, 4.93) | 3.31 (2.88, 3.81) | 1.01 |
| Persons without diabetes | 4.80 (4.21, 5.46) | 3.69 (3.21, 4.23) | 1.11 |
| Persons with diabetes | 11.23 (9.22, 13.62) | 8.77 (7.18, 10.68) | 2.46 |
| **Females** |  |  |  |
| Non-smoker | 2.50 (2.20, 2.85) | 1.91 (1.67, 2.20) | 0.59 |
| Former smoker | 3.02 (2.65, 3.44) | 2.31 (2.01, 2.66) | 0.71 |
| Current smoker | 6.26 (5.61, 6.97) | 4.83 (4.25, 5.48) | 1.43 |
| Statin user | 3.47 (2.58, 4.66) | 2.65 (1.96, 3.58) | 0.82 |
| Statin non-user | 2.50 (2.20, 2.84) | 1.91 (1.66, 2.19) | 0.59 |
| Persons with hypertension | 4.03 (3.50, 4.62) | 3.09 (2.66, 3.58) | 0.94 |
| Persons without hypertension | 2.16 (1.89, 2.46) | 1.65 (1.43, 1.89) | 0.51 |
| Persons without diabetes | 2.50 (2.20, 2.85) | 1.91 (1.67, 2.20) | 0.59 |
| Persons with diabetes | 6.06 (4.92, 7.43) | 4.67 (3.79, 5.74) | 1.39 |
| The 20-year predicted risks (%) of cardiovascular disease-related mortality calculated from logistic regression models. Unless indicated by the stratification variable, these estimates are for a non-smoking male participant, aged 56 years, with a BMI of 25.5, a total daily metabolic equivalent score of 56, with a mean household income of 394 701 – 570 930 DKK/year, 8 – 10 years of education, an alcohol intake of 13 g/day, no prevalent chronic disease at baseline (diabetes, cancer, chronic obstructive pulmonary disease, or chronic kidney disease). | | | |

| **Supplementary Table 5.** 20-year predicted risk of cancer-related mortality for males and females | | | |
| --- | --- | --- | --- |
|  | **Vitamin K_1_ intake** | | **Risk difference**  **(%)** |
|  | **Q1**  **Risk (95% CI)** | **Q5**  **Risk (95% CI)** |  |
| **Males** |  |  |  |
| Non-smoker | 8.13 (7.37, 8.96) | 6.33 (5.70, 7.01) | 1.80 |
| Former smoker | 9.75 (8.88, 10.70) | 7.62 (6.89, 8.41) | 2.13 |
| Current smoker | 17.13 (15.86, 18.47) | 13.62 (12.46, 14.88) | 3.51 |
| **Females** |  |  |  |
| Non-smoker | 6.54 (5.96, 7.17) | 5.07 (4.59, 5.60) | 1.47 |
| Former smoker | 7.87 (7.17, 8.64) | 6.12 (5.53, 6.77) | 1.75 |
| Current smoker | 14.05 (13.01, 15.15) | 11.09 (10.13, 12.13) | 2.96 |
| The 20-year predicted risks (%) of cancer-related mortality calculated from logistic regression models. Unless indicated by the stratification variable, these estimates are for a non-smoking male participant, aged 56 years, with a BMI of 25.5, a total daily metabolic equivalent score of 56, with a mean household income of 394 701 – 570 930 DKK/year, 8 – 10 years of education, an alcohol intake of 13 g/day, no prevalent chronic disease at baseline (diabetes, cancer, chronic obstructive pulmonary disease, or chronic kidney disease). | | | |

| **Supplementary Table 6.** Association between vitamin K_1_ intake and all-cause mortality, stratified by total vegetable intake | | | | | | |
| --- | --- | --- | --- | --- | --- | --- |
| **Vegetable intake tertile** | | **Vitamin K_1_ intake quintiles** | | | | |
|  |  | **Q1** | **Q2** | **Q3** | **Q4** | **Q5** |
| 1  (0 – 124 g/d) | No. events | 3,365 | 1,708 | 614 | 163 | 34 |
|  | HR (95%CI) | ref. | 0.87 (0.79, 0.96) | 0.85 (0.76, 0.95) | 0.87 (0.73, 1.02) | 0.89 (0.63, 1.25) |
| 2  (124 – 204 g/d) | No. events | 340 | 1,138 | 1,405 | 1,100 | 331 |
|  | HR (95%CI) | ref. | 0.82 (0.71, 0.96) | 0.78 (0.65, 0.94) | 0.81 (0.68, 0.95) | 0.76 (0.60, 0.95) |
| 3  (204 – 1,529 g/d) | No. events | 49 | 188 | 563 | 1,168 | 1,917 |
|  | HR (95%CI) | 1.23 (0.93, 1.63) | 1.18 (0.99, 1.41) | 1.15 (1.02, 1.29) | 1.10 (0.98, 1.23) | ref. |
| Hazard ratios (95% Confidence Intervals) for all-cause mortality (n = 14 803) during 23 years of follow-up, obtained from restricted cubic splines based on Cox proportional hazards models. Model 1b adjusted for age, sex, BMI, smoking status, smoking pack-years, physical activity, alcohol intake, social economic status (income), education, and prevalent disease. *Note: the reference level for tertile 3 was set to be Q5 (instead of Q1) due to low numbers of events in Q1 and estimates should be interpreted accordingly. | | | | | | |
